# Long-range non-diffusive spin transfer in a Hall insulator

**DOI:** 10.1038/s41598-018-29323-8

**Published:** 2018-07-19

**Authors:** L. V. Kulik, V. A. Kuznetsov, A. S. Zhuravlev, A. V. Gorbunov, V. V. Solovyev, V. B. Timofeev, I. V. Kukushkin, S. Schmult

**Affiliations:** 1 Institute of Solid State Physics, Russian Academy of Sciences, Chernogolovka, 142432 Russia; 2 National Research University Higher School of Economics, Moscow, 101000 Russia; 3 Max-Planck-Institut für Festkörperforschung, Heisenbergstraße 1, 70569 Stuttgart, Germany; 4 Present Address: TU Dresden, Institute of Semiconductors and Microsystems, Nöthnitzer Straße 64, 01187 Dresden, Germany

## Abstract

We report on optical visualization of spin propagation more than 100 µm. We present an electronic system in a new state of aggregation, the magnetofermionic condensate, in which the lowest-energy spin excitations − photoexcited spin-triplet magnetoexcitons – freely propagate over long distances, in the order of a millimeter, which implies non-diffusion spin transport. Our results open up a completely new system suitable for spintronic devices.

## Introduction

Spin is the intrinsic form of angular momentum carried by elementary particles and quasiparticles. The current revival of interest in long-range spin transfer is supported by high expectations for its use in novel applications based on the manipulation of the spin degree of freedom, within the new technological paradigm of spintronics. Experimental efforts have been directed toward creating materials with large spin diffusion lengths. However, the main spin dissipation mechanisms involve spin-orbit and spin-spin interactions, which are determined by the structural properties of the materials, on which only limited external influence is possible. As a result, the spin diffusion length can range from several nanometers to several tens of nanometers, and sometimes can reach micrometer scales.

The problem of dissipationless spin transport in the solid state has been perplexing minds since the middle of the last century but is yet to be solved. Theoretical predictions have been made on dissipationless spin transport for some materials such as ferromagnetic insulators with easy-plane magnetic anisotropy, zeroth Quantum Hall state of graphene, indirect exciton ensembles in two dimensions, etc.^1–7^. In these materials, the spin carriers are not only electrons but also the collective excitations of the electron system, the magnons^8^, and even so exotic topological magnetic excitations as skyrmions^9,10^. However, most experimental efforts showed spin diffusion length in a very limited range^1^.

**Figure 1:**
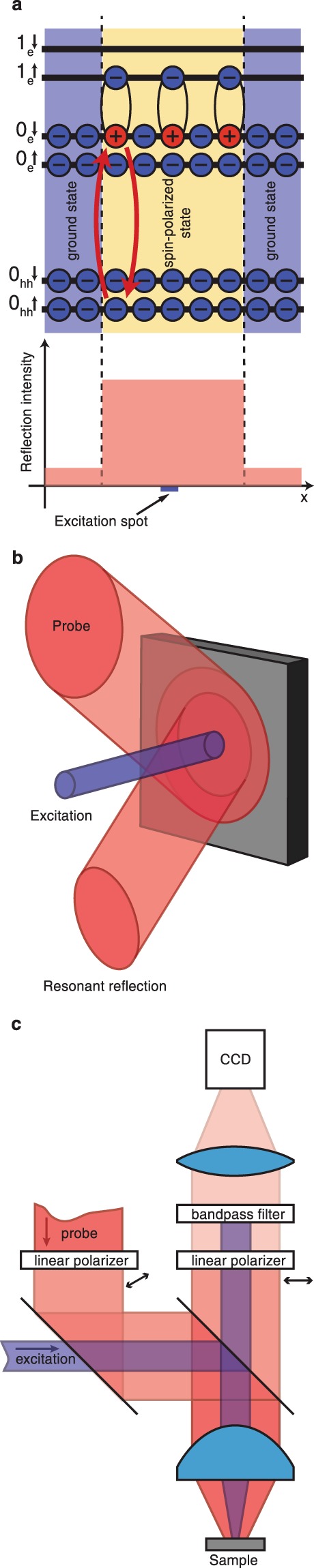
Visualization of spin propagation. (**a**) 2D single-electron energy states in magnetic field. Two red arrows indicate the allowed optical transitions resulting in the resonant reflection which is used to monitor spin system. (**b**) Principle of optical pump-probe method applied to detect spin propagation. The signal of resonant reflectance arises as soon as the spin excitations appear within the broad area illuminated by resonant probe laser beam. (**c**) Simplified scheme of experimental setup for visualization of spin propagation.

In a Hall insulator at filling factor *ν* = 2, the electron Fermi level lies in the cyclotron gap separating the electron states of the zeroth and first Landau levels. The lowest energy spin excitations are the cyclotron spin-flip excitons (CSFEs) composed of an excited electron with flipped spin in the first Landau level 1_e_ and a Fermi hole (electron vacancy) in the zeroth Landau level 0_e_^11^ (Fig. 1a). These spin excitations are electrically neutral quasiparticles, which obey the Bose–Einstein statistics. An electron-hole symmetry is exhibited, wherein the mass and charge (with opposite sign) of the excited electron are equal to those of the effective Fermi hole. Luckily, this symmetry is incomplete − the electron has a *p*-type wave function and the hole has an *s*-type wave function. This creates an opportunity for a finite-temperature Berezinskii–Kosterlitz–Thouless phase transition^12^. The propagation of spin excitations is related to neither the charge transfer nor the mass transfer (the local electron density remains unchanged), but to the energy and spin transfers. The lifetime of cyclotron spin excitons reaches unprecedented millisecond range^13,14^. Therefore, the task of creating a saturated spin exciton gas is experimentally attainable. Аs long as the spin excitons remain in the gaseous phase, the spin diffusion length does not exceed few micrometers, which is in good agreement with the results obtained for other electron systems^1^. As soon as the density of spin excitons in two-dimensional (2D) electron gas reaches a critical value, a phase transition to a new condensed state, *the magnetofermionic condensate*, is observed^14^. Its formation is accompanied by an increase in spin propagation length by at least three orders of magnitude. This is despite the fact that in a 2D electron system the quantizing magnetic field should restrict spin diffusion length^15^.

One of the most challenging problems in the study of spin diffusion phenomena with transport techniques is separating the charge and spin degrees of freedom^16^. We developed a purely optical experimental technique for visualizing the spin transport under the formation of a magnetofermionic condensate. The main idea involves the detection of a single component of the spin exciton, a Fermi hole, by means of photo-induced resonant light reflection (PRR)^13,14^. Here we report results of optical visualization of spin propagation within sample area exceeding 200 µm in size. The main concept of the present experiment is illustrated in Fig. 1b: while creating spin magnetoexcitons in a restricted sample area (excitation spot), we can probe far larger area around, using a defocused resonant laser beam. In the state of magnetofermionic condensate we observe long-range spin exciton propagation, the spatial profile of PRR intensity not described by Gaussian.

## Results and Discussion

**Figure 2:**
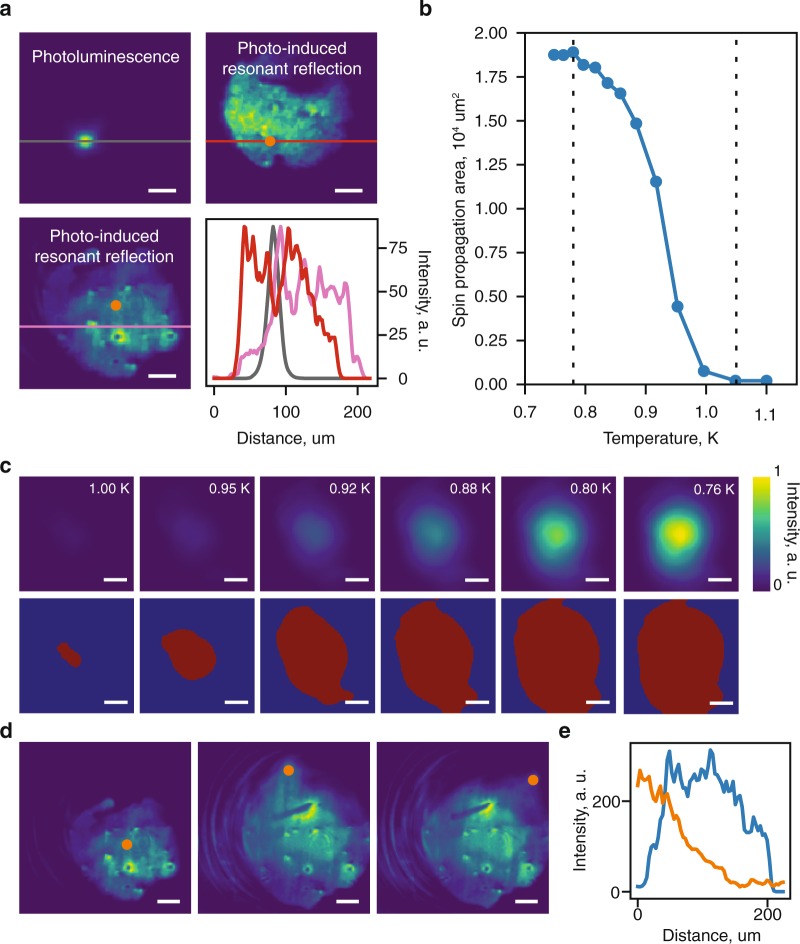
Propagation of spin over macroscopic distances. (**a**) Top left: photo-excitation spot seen in photoluminescence light (0_e_-0_hh_ optical transition). Bottom left and top right: images of magnetofermionic condensate obtained with PRR at excitation in two different sample areas under similar experimental conditions (*T* ≈ 0.5 K). Excitation spot is marked with an orange circle. Bottom right: Cross sections of photoluminescence and PRR intensity along the lines at images (color denotes image). (**b**) Temperature dependence of the spin propagation area. The phase transition to the magnetofermionic condensate is enclosed between two dashed lines. (**c**) Evolution of spin propagation area with temperature. Upper part is averaged and filtered experimental data. Lower part is binarized image with red color corresponding to the presence of magnetofermionic condensate. *P* = 80 mW/cm^2^. (**d**) PRR images of the same sample area obtained at different excitation spot positions marked with an orange circle. Note, that concentric line pattern at left part of the images corresponds to an artifact of an imaging system. (**e**) Cross sections of PRR image (blue curve) and probing light pattern (orange curve) along the same straight line. Everywhere scale bars are equal to 30 μm.

We do not observe an escape of spin excitons from the excitation spot as long as the spin exciton density is less than the critical value for the formation of the magnetofermionic condensate^14^. The excitation spot is directly visualized by photoluminescence corresponding to optically allowed recombination channel of electrons from the zeroth Landau level 0_e_ in the conduction band to the zeroth Landau level 0_hh_ of heavy holes in the valence band (Fig. 1a). The correspondent recombination time is close to 100 ps. Therefore, size of the luminescent spot remains constant at any studied temperature (Fig. 2a, top left). Bearing in mind the spatial resolution of our optical system, we conclude that the spin exciton diffusion length in the studied system does not exceed 2 µm. Calculations made within a single-exciton approximation^14^ give an estimate for the spin exciton diffusion length as a few hundreds of nanometers, which is in reasonable agreement with our experimental data.

When a phase transition into a condensed state occurs, spin excitations start spreading from the excitation spot at macroscopic distances (Fig. 2a). It appears as if the distance of spin propagation over the sample does not have a definite value, but depends on the size of the excitation spot and the excitation power. The spin propagation length shown in Fig. 2d exceeds 200 µm for an excitation spot diameter of 20 µm. This significantly exceeds the values in the currently known experimental data on the diffusion lengths of magnons^8^. With a larger excitation spot area, the spin spreading increases. However, owing to the long-wave fluctuations of electron density in the sample, the propagation area becomes strongly laterally anisotropic. The maximum propagation length measured in our experiments reached 2 mm^14^, and was limited by the size of the studied sample only.

The spin distribution from the edge of the excitation spot toward the edge of the spin propagation spot is not described by a Gaussian distribution. Instead, a spin-density plateau is observed over the entire spin propagation spot (Fig. 2a). Thus, exciton transfer is not a diffusive process. Further, “dark” regions without spin excitations appear within the spin propagation area (Fig. 2a). These are regions where the magnetofermionic condensate is unable to form because of surface defects (micro-scratches, micro-cracks, etc.) destroying the 2D electron layer. Figure 2e proves that features of PRR images are connected with 2D electron system, not probe illumination profile. Taking into account the gigantic propagation lengths of the spin excitations and the non-diffusive character of the spin distribution in space, we claim that the spin propagation in a magnetofermionic condensate is dissipationless. Since the spin propagation length varies with temperature (Fig. 2c), it is possible to either block the spin propagation completely or to permit spin propagation at a precise distance by changing the temperature over a narrow range of ~0.2 K (Fig. 2b).

## Summary

No known material has enabled controllable selection of spin propagation length so far. Therefore, the application of magnetofermionic condensates for spin transfer opens up new opportunities for the manipulation of the spin degree of freedom in solids. It becomes even possible to create a temperature-controlled spin gates and design the spin transistor devices. This assay could be an onset of a new family of temperature-driven spin gates and spin transistors.

## Methods

### Probing Dark Magnetoexcitons

The properties of the explored excitonic ensemble under the conditions of varying experimental parameters such as electron temperature *T*, photoexcitation power density *P*, and magnetic field *B*, were studied by photo-induced resonant reflection (PRR)^13^. The principal complexity of the problem is that cyclotron spin-flip excitons (CSFEs) are “dark” quasiparticles that do not interact with the electromagnetic field. The PRR method enables visualization of spin exciton distribution in the range of photon energies well above the exciton energy, i.e. through dipole-allowed interband optical transitions between discrete Landau levels from the valence band of heavy holes to the electron conduction band (Fig. 1a). Such optical transitions can be used to test separate spin exciton components: the excited electron above the Fermi level and the Fermi hole (electron vacancy) just below. Although spin-flip excitons *per se* are not optically active in the dipole approximation, direct interband transitions from the valence to conduction band are allowed.

The principle of the PRR method is fairly obvious. In a two-level system photo-induced resonant reflection comes to absorption and emission of a photon with energy corresponding to the energy difference between the levels. In Fig. 1a the process is illustrated by two red arrows indicating: (i) transition of an electron from the lower spin sublevel of the zero heavy-hole Landau level in valence band, 0_hh_↑, to the upper spin sublevel of the zero electron Landau level in conduction band, 0_e_↓, with absorption of a resonant photon and (ii) reverse emission of the photon. To enable the process, it is essential that there is an electron in level 0_hh_↑ and a vacant state in level 0_e_↓, i.e. an electron vacancy or a Fermi hole (the red circle with “+” in Fig. 1a). In the Hall insulator with filling factor ν = 2 in the thermodynamic equilibrium state the first condition is fulfilled and the other is not: all the states in the electron Landau level are occupied. For this reason, in the experiment there is no reflection signal for the photons with the indicated energy until the occurrence of non-equilibrium Fermi holes. Since the CSFE consists specifically of a Fermi hole in level 0_e_↓ and an electron in level 1_e_↑, the occurrence of spin-flip excitons can be detected by resonant photon reflection of photons with energy close to that of the corresponding single-electron transition visible in the photoluminescence spectrum. Spin-flip excitons can be also detected by their electron component using transitions from the valence band to initially vacant level 1_e_↑ where non-equilibrium excited electrons occur. For photons with such energy the reflection signal is maximum in the equilibrium state until the occurrence of non-equilibrium excited electrons and decreases with increasing number of non-equilibrium CSFEs.

To create a macroscopic non-equilibrium ensemble of cyclotron spin-flip excitons in the GaAs quantum well, we applied sub-barrier photoexcitation, i.e. light with photon energy less than the bandgap of the barrier compound, Al_x_Ga_1−x_As. The differential PRR spectrum was obtained as the difference between the reflection spectra recorded with the pump laser switched on and off. The study of the PRR signal related to non-equilibrium Fermi holes and/or excited electrons enabled us to make a conclusion about the behavior of the whole spin exciton ensemble^13^. In a specially performed experiment it was checked that PRR signals for Fermi holes in level 0_e_↓ and electrons in level 1_e_↑ exhibit a self-consistent, correlated behavior exactly as described above: the former increases and the latter falls with increasing CSFE concentration. Besides, the kinetic measurements demonstrated identical relaxation dynamics with the pump off^13^. In the experiment it is usually more convenient to proceed with a low signal on the zero level than with a small difference of two large signals. That is why in the experiment we used resonant reflection probing the hole component of the spin-flip excitons. Herewith, it should be noted that it is measurement of PRR intensity for photo-excited electrons that enables us to estimate the concentration of non-equilibrium CSFEs with respect to the total electron concentration in the 2D channel. In this case the possibility exists of correct normalizing to the maximum PRR signal observed in the equilibrium state and corresponding to the maximum possible number of states in level 1_e_↑. At the same time the PRR signal for non-equilibrium Fermi holes in level 0_e_↓ is always observed against some background the amplitude of which is determined by a number of factors that are difficult to estimate quantitatively.

### Samples

The object of the study was a high-quality GaAs/AlGaAs heterostructure with a 35-nm-wide symmetrically doped single GaAs quantum well. The electron concentration in the 2D channel was 2 × 10^11^ cm^−2^ and the dark mobility was above 15 × 10^6^ cm^2^/V·s. At temperatures below 1 K, the spin exciton lifetime exceeded 1 ms^14^. The high quality of the structure and long exciton lifetime were crucial for detection of spin excitons and absolutely essential for observation of the collective effects in the spin exciton ensemble.

The choice of the width of the quantum well was conditioned by two facts. Narrower quantum wells (20–25 nm) do not ensure required high electron mobility, and wider quantum wells (40 nm) do not allow creating a desired degree of disequilibrium. The point is that the relaxation time of non-equilibrium spin-flip excitons reduces rapidly with increasing width of the quantum well owing to flattening of the Coulomb minimum in the dispersion curve of magnetoexcitons^17^. The optimum of quantum well width for creating a non-equilibrium CSFE ensemble at *ν* = 2 is reached at quantum well width of 30−35 nm. The relaxation times of non-equilibrium magnetoexcitons at other integer filling factors turn out to be much shorter (see, for instance^18^). Therefore, for the present it seems to be impossible to create quasistationary non-equilibrium magnetoexciton systems with a concentration of 10^10^ cm^−2^ and above at integer filling factors different from *ν* = 2.

### Experimental Design

The sample of 3 mm × 3 mm in size was mounted inside an insert with liquid ^3^He, which in turn was placed into a ^4^He cryostat with a superconducting solenoid. Samples were studied under quantizing magnetic field of 4.2 T (filling factor *ν* = 2) at temperature range of 0.45–1.5 K. The insert with ^3^He designed for optical measurements with spatial resolution, allowed imaging the sample positioned in the cryostat at a depth of 1.8 m inside a 20-mm diameter thin-walled stainless steel tube through an optical window at the top of the ^3^He insert (not shown in the simplified scheme in Fig. 1c). Two quartz glass lenses: a short-focus aspherical lens, with a focal length of 15 mm and numerical aperture of 0.54 (lens A), and a long-focus spherical lens with a focal length of 275 mm (lens B), both mounted in the tube, – formed a composite high-aperture objective (shown schematically in Fig. 1c by a single lens) with the sample placed near its focal plane.

Two tunable continuous-wave lasers with linewidths of 20 and 5 MHz were employed in the experiment. One served for photo-excitation (optical pumping), and the other − for diagnostics of the electron system (probing) by means of PRR. Also, pumping laser emission was utilized for excitation of photoluminescence. Laser emission was delivered to the cryostat by two optical quartz glass fibers. One of them, a single-mode fiber, was used to transfer the excitation laser beam (the violet beam in Fig. 1b,c), whereas the other, a multimode fiber of 400 μm diameter and numerical aperture N.A. of 0.39, was used to deliver the probing laser beam to the insert (the red beam in Fig. 1b,c). The divergent light beams emerging from the fibers were made parallel by two individual lens collimators. Then the two parallel beams were superimposed in a beam-splitting cube and directed into the insert with ^3^He through the top optical quartz glass window by means of another beam-splitter (semitransparent glass plate). The composite objective inside the insert focused both laser beams onto the sample surface. The size of the focal spot of the probing laser was always much larger than that of the pumping laser. The positions of the laser spots on the sample could be changed independently by adjusting the lens collimators. The laser light reflected from the sample and the luminescence emission from the sample were converted to nearly parallel light beams by the same composite objective, and coupled out through the top optical window. With the help of an extra mirror placed in the path of the light emerging from the cryostat (not shown in Fig. 1c) and a focusing lens the emission from the sample could be coupled into an additional multimode fiber and transferred onto the entrance slit of a grating spectrometer equipped with a liquid-N_2_ cooled CCD camera. The resonant reflection spectrum was recorded by scanning the probing laser wavelength and measuring the intensity of the laser light reflected by the sample. The useful signal of the resonant reflection related to the non-equilibrium Fermi-holes involved in spin excitons was filtered from the probe light reflected/scattered on the surfaces of the sample and optical elements using a pair of crossed linear polarizers installed at the entrance to and at the exit from the cryostat (Fig. 1c). To minimize the heating effects when measuring the PRR spectrum, the pump power on the sample did not exceed 3 μW. The power of the probing laser light was less by two orders of magnitude; thus, it could be considered as introducing no perturbation into the system. The pumping power density in excitation spot was kept typically in the range of ≤ 100 mW/cm^2^ in full correspondence with phase diagram of magnetofermionic condensate^14^.

A magnified image of the sample was obtained by focusing the light emerging from the cryostat window onto the sensitive area of a Peltier-cooled CCD camera using a long-focus microscope objective with a focal length of 190 mm (Fig. 1c). By changing the distances between lenses A and B inside the insert tube and between lens A and the sample, the field of view of the described optical system could be varied from 0.4 mm to 1.3 mm, with the optical resolution decreasing from ≈2 μm to ≈10 μm, respectively. However, due to the low N.A. of this optical system for external light routed through the top optical window the strong vignetting occurred of the probing laser beam. In particular, at optical configuration providing maximal spatial resolution the size of sample area available for testing was limited to 220 μm only (see Fig. 2a).

To visualize the spatial distribution of the PRR signal the line of the probing laser was tuned in resonance with the proper 0_hh_-0_e_ optical transition (Fig. 1a)^13,14^. To cut off the pump laser input from the PRR signal, the light of the probing laser reflected from the sample was passed through a bandpass interference filter (≈1.1 nm FWHM) tuned exactly to the probe wavelength (Fig. 1a).

At minimal temperature *T* ~0.5 K and the fixed pumping intensity *P* the maximal size of spreading spot crucially depended on the size of excitation spot. The observed spin propagation area drastically increased with photoexcitation area. As a result, the useful range of excitation spot was limited by ≤20 μm. In the case of 50 μm excitation spot, for example, the maximal size of spreading area easily exceeded the available 220 μm field of view for PRR images. On the other hand, at fixed *T* the maximal size of spreading spot quickly saturated with *P* (also in correspondence with phase diagram presented in^14^). These circumstances forced us to measure temperature dependence of spin propagation area by permanent registration of PRR images at slowly changing *T* and fixed *P* (see Fig. 2b,c).

Images, obtained by the CCD camera, were computationally analyzed to calculate the spin propagation area (files with raw data and code can be found in^19^ and^20^, respectively). The images were processed by median filter and binarized. The threshold level for cutting-off the PRR signal intensity was chosen to be higher than five standard deviations of residual noise. We specifically note, that variation of the threshold value does not significantly change overall “area-temperature” dependence.

### Data Availability Statement

The datasets generated and/or analyzed during the current study are available at Figshare^19,20^.
