# Author Correction: Long-range non-diffusive spin transfer in a Hall insulator

**DOI:** 10.1038/s41598-018-32561-5

**Published:** 2018-09-18

**Authors:** L. V. Kulik, V. A. Kuznetsov, A. S. Zhuravlev, A. V. Gorbunov, V. V. Solovyev, V. B. Timofeev, I. V. Kukushkin, S. Schmult

**Affiliations:** 1 Institute of Solid State Physics, Russian Academy of Sciences, Chernogolovka, 142432 Russia; 2 National Research University Higher School of Economics, Moscow, 101000 Russia; 3 Max-Planck-Institut für Festkörperforschung, Heisenbergstraße 1, 70569 Stuttgart, Germany; 4 Present Address: TU Dresden, Institute of Semiconductors and Microsystems, Nöthnitzer Straße 64, 01187 Dresden, Germany

Correction to: *Scientific Reports* 10.1038/s41598-018-29323-8, published online 19 July 2018

The original version of this Article contained errors. Affiliation 3 was incorrectly given as ‘Max-Planck-Institut für Festkörperforschung, Stuttgart, 70569, Germany’. In addition, an affiliation was omitted for S. Schmult, which is now listed as Affiliation 4. The correct Affiliation 3 and affiliations for S. Schmult are listed below:

Max-Planck-Institut für Festkörperforschung, Heisenbergstraße 1, 70569 Stuttgart, Germany

Present address: TU Dresden, Institute of Semiconductors and Microsystems, Nöthnitzer Straße 64, 01187 Dresden, Germany

These errors have now been corrected in the PDF and HTML versions of the Article.

